# The Terchloride of Carbon

**Published:** 1864-04

**Authors:** 


THE TERCHLORIDE OF CARBON.—Mr. Bryant informs us that at Guy's Hospital the terchloride of carbon has been employed for many years, and that it was a very favorite remedy of the late Mr. Ashten Key, tracing its employment back, therefore, at least fifteen years. As a lotion it has acquired considerable value, and may be looked upon as a stimulant, and in a measure as a disinfectant. In sloughing and fetid ulcers it is of great use. It may be used in the indolent and weak ulcer with general advantage. The usual strength of the lotion is from ♏xx to ʒss of the drug to an ounce of water. It has an agreeable odor and rapid effect. In cases of gangrene, and in sloughing phagedena, it may be employed in its concentrated form with some confidence, a wound thus affected rapidly taking on a more healthy aspect. Upon the whole, it is a very valuable local stimulant, and in Mr. Bryant's estimation ranks above most of the drugs of that class now in use.—*Med. Times & Gazette.*

